# Odontological Society of Pennsylvania

**Published:** 1894-03

**Authors:** 


### Reports of Society Meetings.

### ODONTOLOGICAL SOCIETY OF PENNSYLVANIA.

THE regular monthly meeting of the Odontological Society of Pennsylvania was held December 9, 1893, at the Hall of the College of Physicians, Thirteenth and Locust Streets, Philadelphia.

The President, Dr. Darby, presided.

On motion of Dr. Kirk the general order of business was suspended.

Mr. William R. Newbold, being introduced by the President, read a paper entitled "The Psychological Aspect of Hypnosis."

(For Mr. Newbold's paper, see page 92.)

Dr. Thomas Fillebrown, of Boston, was called upon to open the discussion.

### DISCUSSION.

*Dr. Fillebrown.*—MR. PRESIDENT AND GENTLEMEN OF THE SOCIETY, —I understand I am expected to speak of this subject to-night in its application to dentistry, and thus give a direction to the discussion more positively towards our own specialty.

It must be evident to every one that hypnotism is a many-sided subject and that it makes all the difference in the world from which side we view it, as to the opinion formed of it, as to our expectations of what it will accomplish, and also of what it will not accomplish. I shall speak only of its sedative and obtundent effects, and in this quiet aspect stage performers, of which we have so many, would not even recognize it. A statue presents many aspects. One sees the statue of Franklin from the front, another from the back, then again another sees the side view. Now, if any one were to give a description of the front of the statue, it would give no idea of it to one who had seen only the back. Then, again, how many-sided is personal character! A delineation of the Saviour scourging the money-changers from the temple gives no conception of Christ bringing the widow's son back to life, or of his weeping over Jerusalem. Scientific subjects present the same difference of views. The law of gravitation when studied in relation to the movements of the heavenly bodies and the existence of the earth appears the beneficent power that it is, but when observed as the power that impels the destructive avalanche or sinks a town when the earthquake opens the earth, it seems the embodiment of evil; but beneficence is the rule and evil the exception. The study of medicine in overdose makes it a system of destruction and death, but studied in its proper and rational administration it becomes for "the healing of the nations." Now, we must not judge opium, ether, chloroform, or nitrous oxide by the extravagant exhibitions that may be made. We must not judge the normal effects of opium by the tale of an opium-eater; nor of ether or chloroform by the extravagant effects produced upon those habituated to their use. We properly judge anæsthetics first by their stimulating and strengthening influence, then by their anæsthetic effects and the benefit we get from them during operations. I wish to make this a practical talk, and consequently I take the practical side of it. I find that human nature is most wonderfully susceptible to suggestion in the wakeful state. The blush has been referred to, so has the pallor of fear. We also realize the wonderful effects of joy, and just the bare suggestion of these states brings them about. So with the absence of fear. You take a little child (or a big child, as to that), and you say, "We are feeling quiet." You are assured yourself. Your own presence is suggestive of courage, and you say, "Indeed, we are not afraid," and the troubled heart is stilled at once; the child walks along with you or, it may be, goes independently at your suggestion without any fear or trembling whatever. I have seen suggestion over and over again quiet circulation where the patient was in a perfectly wakeful state, the circulation being perturbed and uneven; and just the quiet suggestion that the circulation was becoming even brought about the result.

I remember one patient I had who wanted to take an anæsthetic to have a tooth out, but would not hear to the use of hypnotism at all. She would have nothing to do with it, but was terribly perturbed, the pulse 120, and by simply suggesting to that patient that the heart was becoming still and that the pulse was growing less, within three minutes it lessened twelve beats, and in a few minutes more I had removed all her disturbance and she was entirely quiet and breathed the anæsthetic without any resistance or disturbance whatever.

In the hypnotic state this condition is increased wonderfully. The receptivity is increased. That has been described, and I need say nothing more about it. It has been my fortune, I believe, to be among the first to make it practical in its application to dentistry. You know very well that a good many years ago—about 1845—Dr. Esdaile, a surgeon in the English army, while stationed in

Calcutta made a record of nearly three hundred major surgical operations under hypnotic conditions, and the patients were absolutely anæsthetic and knew nothing about it and did not suffer. Now, the Eastern nations are more susceptible to hypnotism than the people of this changing and more temperate clime, and here it cannot be depended on in any great number of cases. It is one only out of about twenty among our people that become somnambulistic. If it were to affect but one patient out of twenty it would be of no good to us. After studying the subject and putting two or three things together, one from Bernheim and one from my friend in Boston, Dr. Osgood, and one or two others, I made up my mind that the lighter forms could be made continuous and would be of use in dentistry. I learned to so use it, and found it even true, and it was a great pleasure to me and has been to a great many patients since. When I first commenced I followed Bernheim's directions and believed the fixation of gaze necessary, and directed the subject to look at my fingers or on some bright object, but later I found it unnecessary, and recently it seems to me that formality of movement or manner is not required, and I can hypnotize them just as readily without as with it. Of the methods I will speak later on.

I will now illustrate by a few cases where suggestion, without any pretence to hypnotism, has proved effective. One was a lady that I operated for within ten days. She has been a patient for a good while, and has no sympathy whatever with hypnotism. She was willing her little boy should be hypnotized, but would not think of such a thing for herself. She was in the other day and had an inferior molar tooth that needed filling. It had been filled previously, and the filling came out because of her utter inability to have the cavity put in proper condition. It needed refilling, but was so sensitive she could not bear to have it touched with the excavator, much less with the engine bur. I commenced deliberately with her, talked to her the same as I am talking to you now. I said, "This tooth is becoming anæsthetized. This dentine is becoming less sensitive. Your fear in regard to it is going away. You will be able to let me cut this without your suffering pain, and you will not mind it." I talked to her in that way and took my engine and went to work and cut it. She did not shrink; she did not suffer. She was so amused at it that she laughed and said, "I know all about this. I know what you are saying, and I acknowledge it does not hurt me, but I cannot understand it." A case like that is wonderfully illustrative to me of the power of suggestion. I cut the cavity just as I wanted it, and she absolutely did not suffer.

Another case occurring within the last ten days was that of a woman who was boarding at an institute for nervous patients and who was nervously depleted. It has been almost impossible for her to have operations performed on her teeth because of the disturbed, restless condition and her fears. Her condition was such that the moment I attempted to do anything upon her teeth she became almost rigid, shrinking away as I touched her teeth so I could hardly get near her. Of course it was of no use to talk hypnotism to that woman. I would not have mentioned it on any account, but I did commence with the same kind of suggestion that I did with the other patient, and the result was that in four sittings averaging nearly two hours each I was able to work along steadily at the teeth and do what I desired. At the last sitting the patient became tired of the monotonous talk, and she said she wished I would not talk so much,—it made her nervous; and the minute I began after that she again straightened up, and I had hard work to finish at the last sitting. She had become thoroughly weary and needed rest. When she comes again I can repeat the same process.

Another case is that of a little girl, much disturbed, crying, and unable to let me touch her teeth. I repeated the same process and had no difficulty whatever.

Another case is that of a patient troubled a great deal with headache. Last spring I cured her of a severe neuralgia and headache. She went out of town for the summer and was to let me know if she had any return of her troubles. After a while she wrote me that she was troubled again with headache, and I replied, saying that it would pass away as soon as she read what I had written, and she wrote saying that it was relieved so quickly after she read my note that it frightened her. That is a repetition of what has been done many times, and has nothing at all new in it.

I often now induce hypnotism by simply closing the patient's eyes, putting my hand on the forehead and bringing it down over the eyes to shield from the light, and simply say to the patient, "You are growing quiet. Your feet and limbs are growing sleepy. Your feet, your hands, your arms, your body, are growing sleepy. Your head is growing heavy and sleepy. Your eyelids are growing sleepy." I find in most cases this is all that is necessary.

You can do the same thing and do it in the same way. I notice here my worthy friend Dr. Jack. You all know what a soft, penetrating voice he has. If he talks to the patient and says, "Now keep quiet under this. You will not fear,—your fears are going away. If you will just relax yourself, as I know you will when you sit down in the chair,—if you will grow heavy in the chair and let all your limbs grow heavy, you will find this will all pass away." And when the patient is in that condition, if he will say, "The tooth is becoming anæsthetized. This dentine is becoming anæsthetized. This will not hurt you,—if it hurts you you will not mind it,—it will not shock you," the condition suggested will be sure to obtain. That creates a smile, I know, but if you will study hypnotism and its phenomena and its application to dentistry you will find many places where it will not only excite your mirth, but it will excite amazement, and you will find that to-day we do not half understand human nature. It has been remarked in the paper that this is one of the faculties of human nature which we are just beginning to recognize and understand. It makes all the difference in the world whether you make a half-hearted statement or a positive one. If you take a patient and are afraid yourself that you are not going to accomplish anything, and say, "I guess this won't hurt, but I am afraid it will," you just excite fear enough so that it will surely hurt; but a positive statement has a positive effect on the human mind. I think myself that the hedging, as I facetiously remarked, "if it hurts you you will not mind it," is more to accommodate the operator than the subject. A great many patients say that if it does hurt it is only at the point of contact, it does not extend over the whole system, and that they feel no shock from it.

Often a suggestion will not apply to a whole cavity at a time. In such cases I anæsthetize it in sections, so to speak. I say, "This is anæsthetized. You will not feel this. I can cut it without hurting you." This will serve for a part. On reaching another sensitive point I say, "Wait a moment and this point will become anæsthetized. It is anæsthetized now. Now the pain is going away,—now I can cut it away." Thus attacking new sections and making the same suggestions, I cut clear around the cavity and complete it. I know by your looks that you are incredulous. I was until I tried it; but I say upon my credit for making honest statements, that I have seen these things over and over again so many times that I cannot resist the conclusion that it is a natural physiological effect and one that we have reason to accept. It is no chicanery and no invention. I wish to enforce upon you all the necessity of repeating these suggestions. I found that operating roused the patient from the condition, and the way I overcame it was by continual suggestion. I keep the suggestion going continually to offset the waking process.

The dangers mentioned by the essayist unquestionably exist. I also recognize that in suggestion we have the remedy which will absolutely and entirely insure against them,—indeed, a veritable "similia similibus curantur."

Bernheim testifies that "hypnotic sleep is as free from harm as is ordinary sleep, and in a long practice I have never seen any harm produced by sleep induced by the Nancy method." (At this time Bernheim had induced hypnosis over ten thousand times.)

Liébeault in thirty years' practice found no trouble whatever.

Heidenhain, of Breslau, says, "Hypnosis under the direction of medical men is harmless, and may do much good."

Drs. Moll, of Berlin, Voisin, of Paris, Tuckey, of London, with a host of others in Europe and America, express the same opinion.

In my practice of two years I have never seen a patient experience any trouble whatever. And I have not yet met any operator who had any intelligent practical knowledge of the subject who has observed the slightest sign of danger or trouble.

The President stated that Mr. Newbold would give a demonstration of hypnotic suggestion, and after that Dr. Daland and Dr. Ottolengui, of New York, would address the Society.

Mr. Newbold, taking the stand, said, "I almost feel that I owe you an apology for offering to show you the phenomena of hypnosis. I do not doubt that many of you are better acquainted with these phenomena than I am myself, and neither need nor care to see them again. I have, moreover, such a cordial detestation of public exhibitions of hypnotism that I do not like to put myself in the position of giving one. I have, however, been pressed to show you the phenomena of which I have spoken, and have consented chiefly because I feel that this audience is actuated by a desire to understand the problems involved and not by any vulgar curiosity. I think it is also due to the two gentlemen who will permit me to hypnotize them to-night to state that I have no mysterious 'control' over them except what they give me. I hypnotize them with their full and intelligent consent, and could not do it without it."

Mr. Newbold, taking the first patient, said, "This patient is very susceptible, and he very readily goes into the state upon mere suggestion."

He then seated the patient in a chair upon the platform, passed his hands over his face, closing the eyes and talking quietly, saying "that he [the patient] could hear nothing except what he was saying to him; that he could hear nobody else; that he could not open his eyes, and that he would wake him up when he was through with him."

Having brought the first patient into the desired state, and leaving him sleeping in the chair, Mr. Newbold introduced the second patient, stating that he was a candidate for the doctor's degree at the University of Pennsylvania, and had been studying psychology; that he was a peculiarly interesting subject because he did not go into a very deep hypnotic state, but remained thoroughly conscious all the time he was asleep. "It is difficult to put him to sleep, and he is a thoroughly good witness of the phenomena of hypnotism. He belongs pretty nearly to the first class, in which the motor system is affected, while the sensory system is but slightly affected."

Seating the patient in the chair, Mr. Newbold gazed steadily at him for perhaps two minutes, swaying his (Mr. Newbold's) head and body backward and forward and up and down, neither operator nor patient relinquishing the gaze into the other's eyes. Mr. Newbold suddenly exclaimed, "Shut your eyes!" This the patient did, Mr. Newbold stroking his face and eyelids for a few moments afterwards.

Mr. Newbold then proceeded to illustrate the production of anæsthesia and contractures by suggestion, showing incidentally that patient B was not as suggestible as patient A.

Addressing the patient, Mr. Newbold said, "You can't feel anything in the right hand." Then rapping it and asking the patient if he felt anything, the patient answered, softly, "No."

"Now," said Mr. Newbold, "you can feel;" and rapping the hand, asked if he felt anything, the patient responding, "Yes."

Mr. Newbold put the patient's hand up, and said to him, "You cannot bend it;" also placed the hands together, saying, "You can't open them;" but in both instances the patient did, in a slight degree, bend the hand and open them.

Going to patient A, Mr. Newbold put his hands together, and said, "You can't open them. Try." The patient did not open them. "Now you can open them," said Mr. Newbold, at which the hands were opened. Mr. Newbold told the patient in a number of different experiments upon the hands that he could or could not feel, and, upon questioning the patient, upon touching the hands, the response always indicated that the sense of touch was in the operator's control.

To illustrate spontaneous catalepsy, Mr. Newbold put up the patient's arm, and it remained up. He then said, "Thomas, you cannot take your arm down." After being suspended for some moments, Mr. Newbold pushed it with considerable force downward, but it still remained supported. Upon directing the patient to let the hand fall, he did so.

Mr. Newbold then gave illustrations of the production of hallucinations by suggestion. Turning to the second subject, B, he said to the patient, "Open your eyes." This the patient did. "You see that private room in the University where we meet Tuesday evenings and discuss psychology?" The patient responded, "Yes." "You see persons in the room, but there is no table. Do you see a table?" Answer: "Yes." Mr. Newbold: "But I tell you there is no table. Do you see a table?" Answer: "Yes." Mr. Newbold here called attention to the fact that he was not able to dissociate the parts of the hallucination, but only to suggest it as a whole.

Mr. Newbold next showed how negative hallucinations are produced. "Open your eyes. I am getting dim and fading away. You can see me dimly. Am I getting dimmer?" Patient: "Yes." "Tell me when I am gone. Do you see anything?" Answer: "Yes."

Mr. Newbold stated that he had sometimes succeeded in blotting out the patient's vision, but only for an instant. "The production of positive hallucinations of vision is not anything remarkable in the case of patient B. He is a good visualizer, and can see anything he wishes at any time. These hallucinations never occur spontaneously, but he can produce them voluntarily."

Mr. Newbold then showed that he could not cause negative hallucinations of hearing in the case of patient B, but could in that of patient A. Mr. Newbold said to patient B, "You are deaf in this ear and can't hear. Do you hear anything?" Putting watch to his ear. Answer: "Yes." Drawing watch away, "Hear anything?" Answer: "No." Putting watch closer, "Hear anything?" Answer: "Yes; a watch."

Going to the first patient, Mr. Newbold said, "Thomas, you can't hear anything except what I say." Mr. Newbold snapped his fingers close to his ear and knocked on the chair close to the patient's head and asked if he heard anything. The response was, "No." "Now you can hear," said Mr. Newbold, and, knocking, said, "Do you hear anything?" The response was, "Yes; a knock."

Mr. Newbold then stated that he would like to show how sensitive the hypnotized patient is to every word uttered by the operator. He raised the right arm of patient A, touched it lightly without saying anything, and waited a few moments. The arm slowly descended and the left arm very slowly rose to about the same height. Mr. Newbold explained that twice, the last time six days before, he had remarked in the presence of the patient, but to a third person, that the effect of touching first the right and then the left arm would be to cause the right to descend and the left to rise. The suggestion had proved effective on both former occasions, and at the time of the second experiment the first was found to have been forgotten. On this third occasion, however, although six days had elapsed since it had last been tried, the subject still remembered that apparently chance remark. The extreme slowness of the movement probably indicated that the suggestion was much weakened by time.

Mr. Newbold then performed an experiment with subject A, designed to show, first, that the hand anæsthetized by suggestion is not truly anæsthetic; second, that the sensations received through it, although not felt as sensations of touch, may serve as *points de répère* upon which to construct complicated hallucinations of vision.

Going to patient A, Mr. Newbold, holding a coat between the patient's eyes and his hand, said, "When I put anything in your hand you will see it on that table over there, but you won't feel it. This hand has no feeling whatever" (putting a knife in his hand). "What do you see on the table?" "Something like a knife." Mr. Newbold held the knife in different directions in the patient's hand, and the responses from the patient were to the effect that he saw it on the table indicated accordingly as it was placed in his hand. The same experiments were made, using a watch instead of a knife.

Here Mr. Newbold woke the patients up, telling them they would feel no disagreeable sensations nor headache; that no one could put them to sleep against their wills, and that they would never go into the state accidentally.

Addressing patient A (after he had waked up), Mr. Newbold said, "Now I shall try to put you to sleep, but don't go to sleep unless you want to. Make up your mind you won't. You must try to stop me." He then tried to hypnotize A for a minute or two, but without success. With this he closed the demonstration.

*Dr. Ottolengui.*—Some years ago I was very much fascinated with the idea of becoming a hypnotizer. That was when I was entirely ignorant of the subject. After I really learned to hypnotize I soon abandoned it in dental practice for several reasons, not the least of which was that it did not seem professional to hypnotize one against his or her will; and I very quickly learned that it would be a disadvantage to me if it got out among my patients that I was in the habit of hypnotizing people, because of the ignorance of the community and the dread of getting into another's power.

You heard Dr. Fillebrown say that at first he used the method of concentrating the gaze upon a bright object, but he had finally abandoned that because he thought it preferable to have the eyes open. Now, while I do not absolutely hypnotize nor attempt to hypnotize, I not infrequently close the eyelids and rotate the eyeballs through the lids when presenting suggestions, because I think that is the quickest way to produce mild hypnosis. Whether true or not I don't know, but my experience seems to substantiate the theory that there are absolutely what has been called hypnogenic zones in the body, and that they very frequently lie back of the optic nerve. Now, you may ask how closing and rotating the eyeballs can have any effect; but I need only ask you to close your eyes and rub them and see if you do not get a sensation of light, and it is that monotony of light-waves which it is claimed will produce hypnosis.

Before I leave the subject of hypnotism itself I would like to state a little of my experience with it and why I abandoned it. In the first place, I found a very small proportion of people were absolutely hypnotic under my influence, and with these few I met some very unpleasant symptoms; patients becoming difficult to control, and in an hysterical condition afterwards. Another curious thing was that patients would not always speak the truth. That is, they pretended to be hypnotic when they were not. Some patients had just enough knowledge of hypnotism to know when I was about to make an experiment upon them, and they were unwise enough to try to fool me into the belief that they were hypnotic. After studying the subject of hallucinations, of which you had a beautiful example here this evening, I formed a test of this kind which has proved quite satisfactory in exposing several persons whom I had previously supposed to be hypnotic subjects. The test is this: take a white glass bottle and suggest to the patient that it is blue; the patient admits it. The patient will admit it whether he is a fraud or honest, because if he is honest the suggestion will make him see it blue; if he is dishonest he knows you mean him to see it blue, so he says it is blue. At that point you have gained nothing. Now, on the third test I take a yellow glass and hold it before the patient's eyes. The malingerer reports that the bottle is yellow. Why? The light rays passing through the white bottle and through the yellow glass makes him see it yellow. He does not know what I am trying to get at. But if the patient is hypnotic he will report that the bottle is green, because it has been suggested to him that it is blue; therefore when the rays pass through a yellow medium it becomes green. I would suggest to a hypnotizer that this is a very pretty experiment to try.

Not only do I believe that there is no person who is not liable to suggestion, but I believe there can be no word spoken which does not carry suggestion with it, in consequence of which we should be very careful what we say to our patients. We see that in the effect a newspaper has on its readers. People who read a certain paper and no others, swear by anything they see in it. The publishers of that paper take advantage of this, especially in a political campaign. Take a Democratic paper. You say the Republicans don't read it, but the Democrat who reads it will go among his Republican friends and use its arguments. He does not realize that that is exactly why they were printed.

Dr. Fillebrown told us that Dr. Esdaile was very successful in Calcutta because the natives are very susceptible. When he tells us of his success in Boston we should certainly recognize the fact that the people of Boston are very much more susceptible than those of our own community. Their literature is impregnated with suggestion; faith-cure, mind-cure, and kindred subjects have been poured down their throats and into their brains until they are ready to believe anything at sight; consequently Dr. Fillebrown has an easier community to work upon than we have.

Cocaine was first introduced to this country through the medium of the newspapers, rather than the medical journals. We were suddenly startled by the information that the drug had been discovered; that it could be injected under the flesh and the part painlessly amputated. I very early experimented with it, and found it did not accomplish all that had been promised for it. At first I almost adopted the idea that cocaine was not a local anæsthetic at all, but I am ready to admit that it does have a therapeutic influence. I remember one day going to a clinic when a patient was shown me who had been unable to have anything done to her teeth until cocaine was administered. She had been given injections of cocaine and a tooth had been extracted without pain. Some of my friends said to me that they wanted to prove to me that cocaine was a valuable agent and that in this case suggestion would not work as well. I said I did not object to being proven in the wrong if I could learn something by it. The patient was a little girl about ten years old, with the remaining superior first molar very much decayed. She said she had had a tooth extracted under cocaine and that it did not hurt. I asked her if she was entirely satisfied that if cocaine were used she would not suffer, and she answered, yes. I told her that I did not believe in cocaine, and asked her if she would have the tooth out with cocaine so that I might see it done. She answered, "Certainly, that is what I came here for." I asked how much her dentist had given her, and she told me three injections of five minims each. I took a similar bottle to the one that had been used before, and giving the three injections, I removed the tooth with all the leisure in the world,—as though I had nothing else to do that afternoon. The child showed no distress or pain whatever. I asked her if she meant me to believe that it had not hurt her, and she replied that there had been no pain whatever. I said, "Gentlemen, that convinces me as to where cocaine stands. What I used was water." They were very much disgruntled, and one of them turned to the patient and said, "Didn't he hurt you? He only used water." She said, "That explains why it hurt me, but I didn't like to say so before." Then they came over to me and told me what she had said. I replied, "The patient is untruthful. Her eyebrows tell the truth: they were not wrinkled a moment whilst I extracted the tooth."

We saw a very pretty experiment here to-night of automatic suggestion, the patient lowering one arm and raising the other. Now, we do not have the same susceptibility in waking patients. We cannot make them quite as obedient as that. In producing an effect by suggestion you must take your patients off their guard; least of all must they suspect that you are trying to influence them by suggestion; consequently I like to add to my suggestions something that they will believe of their own experience. For example, if a patient has a very sensitive tooth, you ask him if he has ever taken chloroform. The answer usually is, "I would like to if only my physician will allow it." I say, "I don't want to give you chloroform, but there is a peculiar thing about chloroform: if you inhale it two or three times, whilst it will not put you to sleep, it will anæsthetize your teeth." The patient says in his mind, "I know chloroform is an anæsthetic. I have no doubt it may influence sensation before it produces sleep; I have every reason to believe the dentist to be honest, because he has never deceived me." That is about what passes through the patient's thoughts. Then take a drop, or as little as you can, and hold it far enough from the patient's nose to be quite out of danger from heart-failure to the most sensitive patient. You ask the patient to tell you when a tickling sensation is felt in the feet, that being the first sign that the drug is at work. A very susceptible patient will feel the coldness coming up at the mere suggestion. Then I say, "Now I will prepare the cavity, and all you have to do is to tell me as soon as you feel any pain ; there is no reason in the world why you should feel any pain." There are but few patients who cannot be affected in this way. Although it is true that you can give the patient chloroform in small doses and obtain an influence or sensation long before unconsciousness ensues, the mere suggestion of chloroform will serve in the majority of cases.

I want to tell of a case that was very curious. A patient said she was losing all her teeth because every dentist she went to sent her to some one else. I found her as troublesome as she was reputed to be. She was very hysterical. The instruments hurt her long before they touched her. I finally lost patience with her and determined that at the next attempt I would prepare the cavity. I had no intention of using hypnotism or even suggestion. I said to her, "This is all nonsense about this tooth hurting you." As soon as she discovered I was determined she became rigid. I was alarmed for a moment and tried to resuscitate her, when it suddenly dawned upon me that she had gone into a mild form of autocatalepsy. I went to work, and after I had completed my filling I said, "It is all over," and she got up instantly. Subsequently she would pass into that cataleptic condition as soon as I closed her eyes. I mention this because she dropped into hypnosis spontaneously.

I find I am getting somewhat of a reputation for not hurting my patients, because for the last two years whenever a patient has said, "My teeth are very sensitive," I say authoritatively, "You will not be hurt; it is the rule of the office never to hurt anybody."

Dr. Judson Daland next took the floor and said,—

"I cannot resist this opportunity of expressing the pleasure I derived from listening to Professor Newbold, particularly in reference to his psychological explanation of the phenomena of hypnotism. This, I take it, is the most difficult problem ever set before man for solution. As to what he has said of the *modus operandi* by which suggestions are carried from the cortex to the periphery, I think in this line we must ultimately find the truth, and to-day we are nearer to it than ever before. The action of suggestions during the waking moments had been exemplified thoroughly by the experiments of Dr. Fillebrown. In the experience of physicians there are many cases where the patients improve as soon as they see the doctor.

"I remember one very interesting case where the patient was hypnotized for the first time. Paralysis of the right arm was suggested with the complete loss of ability to recognize pain. This experiment was readily accomplished, and the patient passed from under observation for a week. At the expiration of this time a second hypnosis was secured, and interestingly enough, the right arm again passed into paralysis and loss of sensibility to pain without suggestion. This was repeated six or seven times at intervals varying from one to three weeks.

"Some remarks have been made regarding the number of cases in a hundred that are susceptible to hypnotism. As to this point, all statistics must, of course, depend upon their classification,—certain phenomena that Bernheim would consider an evidence of hypnotism or Charcot would ignore. I think the three divisions, lethargy, catalepsy, and somnambulism, are, perhaps, the most practical. If the patient is in one of these three states, that state I call hypnotism.

"In the United States the number of patients susceptible is not more than ten or fifteen per cent. My percentages are extremely low,—that may be because I am not a good hypnotizer,—but it is the result of observations upon fifty or seventy patients. Furthermore, results carried on in other countries have yielded better results. In France twenty or forty per cent. may be susceptible; and along with the French certainly must be classified the Spanish and Italian. I do not believe with Bernheim that ninety per cent., nor with Charcot that fifty-two per cent., of all individuals can be hypnotized.

"In reference to the mode of producing hypnotism (of which there are many), I have been rather fond of utilizing one that would separate me from the individual as far as possible. For four years I have used the revolving mirrors of Luys, which I take pleasure in showing you. It is composed of clock-work moving an upright spindle, to which is attached two strips of wood eight inches long and one inch wide, upon each side of which are placed six circular mirrors having a diameter of one inch. The bars revolve in opposite directions. The patient is instructed to look at the central portion steadily, and if susceptible, you will observe hypnotism, sleep, deep lethargy, somnambulism, within twenty minutes. This simply requires concentration of vision upon the revolving mirrors conformed with fixation of the mind upon the idea of sleep. I became interested in this apparatus because of the separation of the operator from the patient, thus eliminating completely all possibility of the influence of the operator's will or of thought transference, and the results have been very satisfactory. I also show another apparatus used by the sportsmen of Germany, which is made of two bars revolving in opposite directions, and containing pieces of various-colored glass placed at different angles. It is placed in the bright sunlight in an open field, and birds are attracted to it by the regularly recurring flashes of light, so that they are easily captured. Luys constructed his apparatus from the instrument. It is well known that animals are hypnotizable, and numerous successful experiments have been made upon chickens, crabs, etc."

*Dr. Madison Taylor.*—I have been waiting to hear if a certain point referred to by the lecturer should be emphasized,—viz., the dangers of too indiscriminate use of these measures. I wish to urge that this point shall be strongly insisted upon in a discussion of this kind. The simple suggestiveness recommended here, the mere hinting to the individual to become anæsthetic, is a simple process readily demonstrable by many.

This, whether used by a properly-instructed operator or novice, may cause some possible mischief, if in no other way, by at least misunderstandings. This was brought out clearly by the lecturer, but not nearly enough in the discussion. It is often most tempting to induce a suggestive domination in a patient, but while securing this effect there may be too large a revelation of things that should remain hidden, which we have no right to discover.

Partial hypnosis offers many useful possibilities, and is frequently most useful, but entails grave responsibilities and some dangers not lightly to be ignored.

Dr. Newbold closed the discussion, reviewing the remarks of the speakers and thanking the audience for their attention.

Adjourned.

